# Complete Sequencing and Chromosome-Scale Genome Assembly of the Industrial Progenitor Strain P2niaD18 from the Penicillin Producer *Penicillium chrysogenum*

**DOI:** 10.1128/genomeA.00577-14

**Published:** 2014-07-24

**Authors:** Thomas Specht, Tim A. Dahlmann, Ivo Zadra, Hubert Kürnsteiner, Ulrich Kück

**Affiliations:** a Sandoz GmbH, Kundl, Austria; b Christian Doppler Laboratory for Fungal Biotechnology, Lehrstuhl für Allgemeine und Molekulare Botanik, Ruhr-Universität Bochum, Bochum, Germany

## Abstract

*Penicillium chrysogenum* is the major industrial producer of the β-lactam antibiotic penicillin. Here, we report the complete genome sequence of the industrial progenitor strain *P. chrysogenum* P2niaD18 in a chromosome-scale genome assembly. P2niaD18 is distinguished from the recently sequenced *P. chrysogenum* Wisconsin 54-1255 strain by major chromosomal rearrangements leading to a modified chromosomal architecture.

## GENOME ANNOUNCEMENT

The ascomycetous fungus *Penicillium chrysogenum* is the major industrial producer of the β-lactam antibiotic penicillin. All industrial strains are descendants of the wild-type strain *P. chrysogenum* NRRL 1951, which was isolated from a cantaloupe stem. Based on NRRL 1951, strains were obtained by X ray and UV mutagenesis, including *P. chrysogenum* Wisconsin 54-1255 and its related former industrial strain *P. chrysogenum* P2 (ATCC 48271) (1). Compared to NRRL 1951, P2 shows a >85-fold increased penicillin titer and was used for conventional mutagenesis to construct P2niaD18, a nitrate reductase-deficient derivative (2).

Here, we report the complete genome sequence of the improved penicillin producer strain P2niaD18 in a chromosomal assembly, which was carried out using an Illumina HiSeq 2000-based whole-genome shotgun strategy. We demonstrate that P2niaD18 contains two copies of the penicillin gene cluster, which are located as a tandem repeat. Further, we verify two major chromosomal rearrangements compared to the genome of Wisconsin 54-1255 leading to a modified chromosomal architecture and nitrate reductase deficiency.

To obtain a high-quality genome sequence of P2niaD18, we improved the genome assembly of Wisconsin 54-1255 (3) as follows: 179 of 195 existing gaps inside the Wisconsin 54-1255 genome sequence were individually closed by comparing the Wisconsin 54-1255 sequences with our genome data from P2niaD18 (see below). Assembly and editing of the Wisconsin 54-1255 genome were performed using the Staden package (4).

By manual *in silico* assembly of the contigs, we obtained 5 scaffolds representing 4 chromosomes and the circular mitochondrial genome of Wisconsin 54-1255. The organization of the four reconstructed Wisconsin 54-1255 chromosomes (I, 10,350,089 bp; II, 9,488,591 bp; III, 6,943,310 bp; IV, 5,586,572 bp) is confirmed by chromosomal size measurements previously done with pulsed-field gel electrophoresis (5).

The whole-genome shotgun sequencing of P2niaD18 delivered ~37 million paired-end reads (100 nucleotides) with a 366-bp average paired-end distance. To reconstruct the complete genome sequence of P2niaD18, a Burrows-Wheeler alignment (6) was performed. By manual inspection and resequencing of PCR amplicons at recombination sites, we were able to identify two chromosomal rearrangements, which led to translocations between chromosomes II and III and chromosomes II and IV. While both translocations result in a modified chromosomal architecture, translocation between chromosomes II and III causes nitrate reductase deficiency by splitting up the nitrate reductase-encoding gene *niaD* (Pc13g11410). In addition, chromosome I shows a tandem repeat duplication of the penicillin gene cluster. These translocations are mainly responsible for altered chromosomal sizes in P2niaD18 (I = 13,597,116 bp, II = 10,455,537 bp, III = 5,401,030 bp and IV = 3,043,715 bp). Using Augustus and tRNAscan-SE (7, 8), 11,839 open reading frames (ORFs) and 188 tRNA genes are predicted for the nuclear genome of P2niaD18.

### Nucleotide sequence accession numbers.

This whole-genome shotgun project has been deposited at DDBJ/EMBL/GenBank under the accession no. JMSF00000000. The version described in this paper is the first version, JMSF01000000.
